# Safety and efficacy of botox injection in alleviating post-operative pain and improving quality of life in lower extremity limb lengthening and deformity correction

**DOI:** 10.1186/1745-6215-8-27

**Published:** 2007-09-28

**Authors:** Reggie C Hamdy, Kathleen Montpetit, Joanne Ruck-Gibis, Kelly Thorstad, Ellen Raney, Michael Aiona, Robert Platt, Allen Finley, William Mackenzie, James McCarthy, Unni Narayanan

**Affiliations:** 1 Orthopaedics. Shriners Hospital for Children, 1529 Cedar Avenue, Montreal H3G 1A6, Canada; 2 Occupational Therapy, Shriners Hospital for Children, 1529 Cedar Avenue, Montreal H3G 1A6, Canada; 3 Physical Therapy, Shriners Hospital for Children, 1529 Cedar Avenue, Montreal H3G 1A6, Canada; 4 Nursing Unit, Shriners Hospital for Children, 1529 Cedar Avenue, Montreal H3G 1A6, Canada; 5 Orthopaedics. 1310 Punahou Street. Honolulu, Hawaii 96826-1099, USA; 6 Orthopaedics. Shriners Hosptial for children. 3101 S.W. Sam Jackson Park Rd, Portland, Oregon 97239-3095, USA; 7 Biostatistics, Research Institute of Montreal Childrens Hospital, 2300 Tupper St., Montreal QC H3H 1P3, Canada; 8 Pediatric Pain Management Service, Isaac Walton Killam Health Center, 5850 University Avenue Halifax NS, Canada; 9 Orthopaedics. Alfred I. duPont Hospital for Children 1600 Rockland Road, Wilmington, DE 19803-3607, USA; 10 Orthopaedics. Shriners Hospital for Children, 3551 North Broad Street, Philadelphia, PA 19140, USA; 11 Orthopaedics. Hospital for Sick Children. 555 University Avenue, Toronto, Ontario M5G 1X8, Canada

## Abstract

**Background:**

Distraction osteogenesis is the standard treatment for the management of lower limb length discrepancy of more than 3 cm and bone loss secondary to congenital anomalies, trauma or infection. This technique consists of an osteotomy of the bone to be lengthened, application of an external fixator, followed by gradual and controlled distraction of the bone ends. Although limb lengthening using the Ilizarov distraction osteogenesis principle yields excellent results in most cases, the technique has numerous problems and is not well tolerated by many children. The objective of the current study is to determine if Botulinum Toxin A (BTX-A), which is known to possess both analgesic and paralytic actions, can be used to alleviate post-operative pain and improve the functional outcome of children undergoing distraction osteogenesis.

**Methods/Design:**

The study design consists of a multi centre, randomized, double-blinded, placebo-controlled trial. Patients between ages 5–21 years requiring limb lengthening or deformity correction using distraction will be recruited from 6 different sites (Shriners Hospital for Children in Montreal, Honolulu, Philadelphia and Portland as well as DuPont Hospital for Children in Wilmington, Delaware and Hospital for Sick Children in Toronto, Ont). Approximately 150 subjects will be recruited over 2 years and will be randomized to either receive 10 units per Kg of BTX-A or normal saline (control group) intraoperatively following the surgery. Functional outcome effects will be assessed using pain scores, medication dosages, range of motion, flexibility, strength, mobility function and quality of life of the patient. IRB approval was obtained from all sites and adverse reactions will be monitored vigorously and reported to IRB, FDA and Health Canada.

**Discussion:**

BTX-A injection has been widely used world wide with no major side effects reported. However, to the best of our knowledge, this is the first time BTX-A is being used under the context of limb lengthening and deformity correction.

**Trial Registration:**

NCT00412035

## Background

### 1. Lengthening and Deformity Correction

Lower extremity limb deformity and length discrepancy can occur secondary to congenital anomalies (such as fibular hemimelia or Blount's disease) or traumatic, infective or neoplastic events leading to partial or total physeal arrest. If the discrepancy exceeds 3 to 5 cm, then equalizing limb length would be the ideal treatment [1].

Since the first bone lengthening procedure performed on a human patient by distraction was reported by Codivilla in 1905 [2], numerous techniques for lengthening bones and correcting deformity have been developed, but these were fraught with unacceptably high complication rates [3]. Then the Russian orthopaedic surgeon Gavriel Ilizarov developed new principles in limb lengthening. Working in the small Siberian community of Kurgan, he found that after a delay of a few days following osteotomy, bone and soft tissue would regenerate when subjected to slow and gradual distraction. He called this the "*Law of Tension Stress" or Distraction Osteogenesis (DO) *[4,5].

The treatment protocol, according to the Ilizarov distraction osteogenesis principle [4,5] consists of an osteotomy of the bone followed by a latency phase of 5 to 7 days. This is followed by the actual lengthening or distraction phase which is continued until the desired amount of distraction is obtained. The optimum amount of daily lengthening has been found to be 1.0 mm, divided into 4 increments. For example, 5 cm of lengthening will require 50 days of distraction. This is followed by the consolidation phase. This is the period for which the fixator has to be left in place, until the newly created bone between the osteotomy ends becomes biomechanically strong enough to withstand the stresses of mechanical usage. This consolidation phase is very long and takes approximately 1 month for every cm lengthened. For example, a lengthening of 5 cm will require 5 months in the fixator.

#### Problems of Distraction osteogenesis

Limb lengthening and deformity correction using the Ilizarov technique [4,5] yields excellent results in most cases [6-10]. However, the technique has numerous problems [11,12] and is not well tolerated by many children. Some of these problems include medical complications such as pin site infections, pain, muscle-related complications (including contractures, joint stiffness and subluxation), oedema, osteoporosis of the lengthened segment, and problems with the bony regenerate such as premature fusion, delayed or non-union. Psychological disturbances may occur during treatment and may necessitate treatment. In addition, the financial burden to the family and the health institution could be significant.

Numerous attempts have been and continue to be made in order to minimize these complications by attempting to accelerate the formation of new bone at the distracted zone [13,14], so that the fixator could be removed at an earlier date. However, to the best of our knowledge, none of these studies focused on pain and muscle spasm or contractures in an attempt to improve the outcome of the lengthening procedure. We were able to find only one full report addressing pain issues in limb lengthening [15]. In this report, the type and amount of pain varied according to the stage of the lengthening process, and occurred throughout the entire process, which again emphasizes the importance of pain as a major component of limb lengthening procedures. Many aspects of the limb lengthening or deformity correction process contribute to pain, including [15]:

1. In the early stages, the corticotomy and incision sites are painful.

2. Acute post-operative oedema contributes to pain.

3. Gradual distraction increases soft tissue tension, resulting in stretching pain sensation from the muscles.

4. Pin sites often become infected.

5. Physiotherapy in the form of range of motion exercises and gait training may intensify already existing pain.

#### Effect of lengthening or correction on muscles

Skeletal muscles have a remarkable capacity for adaptation to gradually increasing limb length or decreasing deformity. Several mechanisms of muscular adaptation to increasing length during distraction osteogenesis have been described [16,17]. The most important one is the addition of new serial sarcomeres to lengthen existing muscle fibers and to prevent sarcomere elongation [16,17], which is very important for maintaining muscular function. However, it has been shown that bones and muscles do not lengthen at the same rate [16]. When the degree of muscle – tendon adaptation during limb lengthening or deformity correction is not enough to adequately accommodate the increase in bone length, muscle related complications such as joint compression, bone growth retardation, contractures and subluxation may occur. Intensive physiotherapy, braces, readjustment of the fixator and even further surgical intervention may then be required.

Another interesting issue to raise is whether or not lengthening causes damage to skeletal muscle? Several studies have shown that lengthening causes structural changes in muscles. Signs of partial muscle denervation during extremity lengthening in humans have been documented by electromyography (EMG) findings [18,19]. Whether this muscle damage documented by EMG findings recovers or not, is controversial. Some authors believe in a permanent effect of extremity lengthening on the neuromuscular tissues [20], while others believe that this damage is temporary as evidenced by the appearance of multiple polyphasic potentials [21]. Some authors [21,22] also found in a canine model of distraction osteogenesis, selective atrophy of Type II muscle fibers (and to a lesser extent Type I) and areas of muscle fiber necrosis during the distraction period. They attribute the Type II muscle fiber atrophy to muscle disuse. These events are followed by increased muscle fiber density secondary to muscle neogenesis.

The question then arises: Can BTX-A injection minimize or even prevent muscle related complications and can it prevent muscle damage during limb lengthening or deformity correction?

### 2. Botulinum Toxin A

Botulinum toxins are extremely potent, naturally occurring poisons. These toxins are fermentation products of the anaerobic spore-forming bacterium *Clostridium botulinum*. Eight immunologically distinct botulinum serotypes have been identified [23]. Of these, seven serotypes – A, B, C1, D, E, F, and G – are neurotoxins. These toxins cause flaccid paralysis by blocking acetylcholine (Ach) release, which is required for muscle contraction, at the neuromuscular junction. Therefore, therapeutic benefit may be obtained by exploiting the pharmacologic properties of carefully administered regional application of this purified neurotoxin.

Types A and B are the only types used in clinical practice. Type A is available in the United States as BTX-A (Allergan, Inc., Irvine, Ca.) and in several other countries as Dysport (Ipsen Ltd., Berkshire, UK). Type B is available in the United States as Myobloc (Elan Pharmaceuticals' San Diego, CA) and in European countries as NeuroBloc (Elan Pharmaceuticals, San Diego, CA). Botox is available in a vial that contains 100 units of toxin. The product is shipped from the manufacturer on dry ice and is stored either in the refrigerator at 2 to 8°C or in the freezer at -5°C. Frozen vacuum-dried toxin is reconstituted with 0.9 ml normal saline to various concentrations usually 2.5 to 10 units/0.1 ml.

Although there are only four indications approved by the FDA for the clinical use of BTX-A (cervical dystonia, strabismus, facial hemispasm and blerephorospasm) [24-26], the use of BTX-A has rapidly expanded beyond these approved disorders and includes a variety of clinical conditions where BTX-A is now being used both for its neuromuscular and analgesic effects [27-45]. The explosion of interest in the biology and clinical applications of BTX-A is reflected by the number of publications in the last few years on the subject. A review of the literature (Pubmed) revealed 5200 entries under the word botulinum injections.

#### Dosing parameters

The volume and concentration of BTX-A have been discussed at the American Academy of Cerebral Palsy and Developmental Medicine (AACPDM) meeting in September 2003. Doses of 20 units/kg/day were reported [46]. It was suggested that decreasing the concentration and increasing the volume injected would be more beneficial. However, a larger volume injected may have the side effect of wider infiltration [47,48].

In two very interesting and most recent studies on the safety profile and efficacy of BTX-A used in Europe (Dysport) in children with spasticity, Bakheit et al, [49,50] reviewed the use of BTX-A in 758 children in 17 different European Centres. The doses of BTX-A ranged from 5 to 18 units/Kg/day. Interestingly, they found that a dose of 10 units/Kg/day gave the optimal clinical results. Furthermore, their data suggest that BTX-A in excess of 1000 units does not improve the therapeutic response and may increase the risk of adverse events. (However, although BTX-A and Dysport are both Botulinum toxin type A, their doses cannot be compared).

#### Median Lethal dose

is estimated to be 3000 units of BTX-A for a 70-kg adult [23].

#### Safety, Risks and adverse effects

BTX-A has been in use as a therapeutic agent since the late 1970s, and has been shown to be a remarkably safe drug when used under medical supervision [51-61]. Since the mechanism of action of BTX-A is so specific, non-neuromuscular side effects are uncommon and systemic effects are very rare [23]. Side effects include a flu-like syndrome that is generally short-lived [62], dysphagia, and dry mouth. Unintended weakness of the muscle injected or of nearby muscles could also be a negative effect. Other side effects include muscle soreness, rash, headaches, light-headedness, fever, chills, hypertension, diarrhea and abdominal pain [23]. In their review of 758 children with chronic spasticity, Bakheit et al, [49,50] reported a 7 % incidence of adverse effects. Another important finding of their study was that the total dose of the toxin per treatment session, rather than that calculated on the basis of body weight, correlated with the incidence of adverse events and also functional improvement or deterioration.

#### Antibody formation

All botulinum neurotoxins are proteins and therefore immunoresistance may develop secondary to formation of blocking antibodies. The incidence of antibody-mediated resistance in long-term treated patients ranges from 3 to 23%, depending on the patient sample, treatment regimen and toxin preparation [51]. With the new BTX-A formulation, (5 nanograms of neurotoxins per 100 units that were introduced in 1997, less than 3 % of patients will develop antibodies [51]. In a most recent study [24] evaluating 130 patients treated for cervical dystonia, 42 had the original BTX-A used before 1998 (25 ng protein/100 units) and 119 had the current BTX-A injection (5 ng protein/100 units). None of the 119 patients who received the new BTX-A injection developed antibodies while 9.5% of those who received the old formulation developed antibodies.

Antibodies against the toxin are presumed to be responsible for most cases of resistance [51]; however, other potential risk factors for immunoresistance include genetic predisposition, frequency of administration, and possibly prior resistance to other botulinum toxin serotypes [24]. Resistance may be minimized by using the lowest effective dose with at least 3 months interval between injections [51]. Patients who develop resistance to one serotype may benefit from a switch to a different serotype [51].

#### Administration

One ml "tuberculin" syringes with 27 – 32 gauge needles are used for the injections. Electrical stimulation or ultrasound may be useful techniques to identify selected muscles during BTX-A therapy but are rarely used. Most centres do not use general anesthesia for the injections [50].

#### Mechanism of action

Botulinum toxins are known to have at least 3 mechanisms of action: paralytic, anti-secretory and analgesic (anti-nociceptive).

##### A. Paralytic effects

Botulinum toxin is a pre-synaptic neuromuscular blocking agent. It produces temporary chemical denervation by blocking the release of acetylcholine, thus, causing muscle relaxation. BTX-A acts on all cholinergic nerve terminals, including those of motor neurons, preganglionic sympathetic and parasympathetic neurons and postganglionic parasympathetic nerves. The molecular mechanism of inhibition of acetylcholine release is a multistep process that has been very well described [51].

Muscle weakness occurs within a few days to 1 week after local injection, peaks within 2 weeks for several weeks, and then plateaus in milder form (the desired clinical effect) before gradually returning to baseline [51]. The effects of BTX-A are dose related. Recovery from the toxin-induced paralysis involves resprouting of terminals from the axon, followed by slow recovery of the neuron's ability to release acetylcholine. The clinical effects last 3 to 4 months after each injection [51].

##### B. Anti-secretory effects

BTX-A blocks parasympathetic nerve-induced secretion and may be useful in conditions of palmar and plantar hyperhydrosis [40].

##### C. Analgesic (anti-nociceptive effects)

The analgesic effects of BTX-A are very intriguing and were first reported in 1985 in a pilot study of BTX-A treatment for cervical dystonia, characterized by abnormal, involuntary neck and shoulder muscle contraction and often resulting in significant disabling pain [63]. It was noted in that study that the most marked benefit of BTX-A injections was pain relief. Since then, numerous reports have confirmed the analgesics effects of BTX-A.

The association between BTX-A and pain relief was originally thought to relate only to its effect on muscle contraction. However, the analgesic effects of BTX-A could not be entirely explained by its muscle relaxation effects. Several studies suggest that other pathways may also play a role in the analgesic effects of BTX-A. The strong suspicion that BTX-A has an anti-nociceptive action independent of its paralytic effect developed because of several clinical observations. First, in conditions of pathologic muscle overactivity (dystonia and spasticity), pain relief often begins quickly before weakness, seems greater than the degree of weakness, and outlasts the weakness [63]. Second, BTX-A has been shown to cause analgesia without weakness in an animal model of neuropathic pain [64]. Third, BTX-A has been shown to block the formalin-induced release of glutamate in the paws of rats [65] and it is known that glutamate mediates the release of neurotransmitters substance P and calcitonin gene related peptide (CGRP) which play a role in transmission of pain. BTX-A also directly blocks the release of substance P and CGRP [66,67]. Therefore, it seems that BTX-A exerts its analgesic effects through several pathways:

##### i. Analgesic effect secondary to muscle relaxation

Many chemicals, including bradykinin, serotonin, potassium, prostaglandin E2, and several neuropeptides such as substance P, glutamate and CRGP can sensitize muscle nociceptors. Because activation of these nociceptors may be related to the degree of contraction of a muscle, BTX-A may ameliorate pain simply by reducing the extent of muscle spasm or contraction and, therefore, uncouple this process [30]. Also, BTX-A may decrease pain due to muscular spasm, by decreasing distortion of structures attached within the muscles and also by decreasing the compression of nerves as they pass within the muscle [68].

##### ii. Analgesic effect secondary to inhibition of peripheral sensitization

BTX-A may have a direct effect on non-cholinergic neurons and reducing peripheral sensitization. BTX-A inhibits the release of the nociceptive or inflammatory neuropeptides substance P [66,67], and CRGP, directly or indirectly, by inhibiting the release of glutamate, which in turn stimulates the release of substance P (peripherally and centrally) and CRGP. Substance P is a neuropeptide that plays a role in pain perception, vasodilatation, and neurogenic inflammation.

##### iii. Analgesic effect secondary to inhibition of central sensitization

Some studies suggest that BTX-A is transported by axons to the CNS after intramuscular injection [68]. Many neurotransmitters are released in vesicles by exocytosis mechanisms that are dependent upon the SNARE proteins, which are the target of botulinum toxins. Therefore, BTX-A blocks the release of more neurotransmitters than ACh, and has been shown to affect neurotransmission in CNS tracts that are involved in pain transmission or modulation [68]. Changes in the central sensory system "neuroplasticity" secondary to pain stimuli could also be affected by BTX-A [67].

##### iv. Analgesic effect through alteration of autonomic function

Blood flow is clearly related to inflammation and ischemic pain and is probably involved in the sensitization of nociceptors. Autonomic neurons alter regional blood flow by controlling the smooth muscle walls of small arterioles. BTX-A may block some of the autonomic vascular control functions and at the same time may alter the release of a variety of non-ACh agents that also affect blood flow. Finally, autonomic function mediated by the release of ACh is associated with both emotional behaviour and stress through complex CNS circuity. BTX-A may alter the global perception of pain and or the patient's overall response to pain through this linkage [67].

Although the analgesic properties of BTX-A could have tremendous potential in a wide variety of clinical problems [69-71]], we were able to find only one study on the effects of BTX-A in relieving post-operative pain in orthopaedic procedures and none in the context of limb lengthening. In a double-blinded, placebo controlled trial [72], Barwood et al. reported profound anti-nociceptive activity of BTX-A injection when administered prior to adductor-release surgery in children with cerebral palsy. The analgesic effect was so dramatic that the trial was terminated early. Children treated with BTX-A had a reduced need for narcotic analgesics, were discharged earlier, and had better outcomes than the placebo group.

The objective of the current multi centre clinical study is to assess if the use of Botox does alleviate pain for children in the early post op period during distraction osteogenesis. This protocol addresses the issues of both pain and muscle spasm through the use of BTX-A injections.

## Methods/Design

The design chosen to test our hypothesis and answer our specific aims is a multi-center prospective, randomized, double-blinded, controlled trial. The pilot study successfully demonstrated that recruitment, randomization and data collection are feasible. In addition, the existence of only one published report in the English literature dealing with post-operative pain in children undergoing limb lengthening [15] and the absence of any data on the use of BTX-A injection for relief of pain in cases of distraction, propel us to proceed with this design.

### 1. Sample and Duration of Study

Subjects will be recruited at each site from patients requiring surgery for limb lengthening or deformity correction using distraction with a fixator. The 6 sites participating in the study include Shriners Hospital for Children in Montreal, Honolulu, Philadelphia and Portland as well as DuPont Hospital for Children in Wilmington, Delaware and Hospital for Sick Children in Toronto, Ont. Based on the historical data at each site for 2003–2006, estimations were made of potential participants for each site. The mean number of children treated for this problem at each participating site varies from 8 to 30 for a total of approximately 75 potential participants per year for the 6 sites.

The total sample size expected to meet the inclusion criteria is approximately 150 subjects to be recruited over 2 years (See section 10 for a detailed power calculation). Subjects will be randomized to either an injection of BTX-A (BTX-A group) or normal saline (control group) and both will continue with standard post-operative nursing care and rehabilitation. Patients undergoing this particular intervention are closely followed in the outpatient clinic after discharge. At each visit they routinely meet the surgeon, the Care Coordinator, and the physical therapist. The site coordinator will track the participants and ensure that the standardized assessments are administered at the appropriate visit.

Subjects will be enrolled during the first 24 months of the 3-year project. Each patient is followed for up to 12 months with data collected longitudinally over this period. The last patients enrolled in year 2 will be followed for 9–12 months into year 3. No recruitment will occur in year 3, however, follow-up on the final patients enrolled in year 2 will continue into the final months of year 3. Analysis will commence in year 3. Depending on the timing and number of participants enrolled in year 2 a continuation to year 4 may be required for final statistical analysis.

#### Inclusion Criteria

1. Age: 5 to 21 years.

2. Etiology of the deformity: congenital or acquired.

3. Amount of lengthening or deformity correction: any amount.

4. Site of lengthening or deformity correction: lower extremity, unilateral single segment.

5. Type of fixator: circular or uniplanar.

#### Exclusion Criteria

1. Children younger than 5 years of age.

2. Associated neuromuscular conditions that may hinder weight bearing.

3. Individuals on aminoglycosides, as aminoglycosides can potentiate the effect of Botulinum toxin A.

4. Neurofibromatosis

#### Stratification by patient characteristic

Participants will be stratified according to three indications for treatment: simple lengthening, lengthening with deformity correction, and soft tissue correction. The amount of lengthening anticipated will be calculated both as an absolute value in cm as well as a percentage of the length of the bone to be lengthened. Amount of deformity correction will be calculated in number of degrees. Participants will be stratified to two categories according to the severity of the limb length discrepancy or deformity correction: < 20% or > 20% of the pre-operative values.

#### Potential Confounding Variables and Other Measurements

Information on infections will be recorded. All relevant concomitant medications will be recorded. These variables will be considered in exploratory analyses.

### 2. Randomization

The randomization occurs at each site according to the procedure successfully used during the pilot. The bio-statistician consultant will generate a computerized block randomization schedule for each site ensuring an equal number of subjects in each group at each site. The coordinating site will provide each site with a set of sealed envelopes containing the randomization code for each patient, which will be opened only by the individual preparing the syringe. The patients will be randomly assigned to either the treatment group or the placebo group. Concealment of randomization will be enforced, so that individuals enrolling patients will be unaware of whether the next patient will be randomized to treatment or control [73,74].

### 3. Masking

As the injection will be done intraoperatively, the patient will be blinded. The treating surgeon will be given a syringe for the injection and the surgeon will not know whether the syringe contains BTX-A or placebo. Parents, nursing staff, research, and rehabilitation staff are also blinded to group assignment. All the personnel involved in the assessments and analysis of the data will be blinded. Only the pharmacy or OR nurse preparing the syringe is aware of the group assignment.

### 5. Ethical Approval

Ethical approval was obtained from Shriners Hospital for Children Headquarters Medical Research Office and from 6 local Institutional Review Board (IRB). The research proposal will be explained to the patient and his or her family. Signed informed consent and assent will be obtained.

### 6. BTX-A Injection Protocol

#### i. Timing of the injections

The surgical procedure will be performed in the usual manner. The injection will be performed intraoperatively under general anaesthesia at the conclusion of the surgical procedure just prior to extubation to avoid any potential effects of the depolarizing agents used at the time of induction. BTX-A will be given only once (during the surgery) and will not be repeated. The advantages of giving the injection during the surgery (versus before or after) are twofold: First it will allow the analgesic action of BTX-A to take effect immediately (which is desired), while the paralytic action of BTX-A will take about 7 to 10 days to take effect (coinciding with the start of distraction). Second, the patient will be blinded and will not know whether he has or has not received the injection.

#### ii. Dosage

- The dosage will be 10 units per Kg. not to exceed 400 Units

- The maximum will be 50 units per injection site

- Maximum dose of 5 Units/kg per large muscle group not to exceed 200 Units

- Maximum volume of 1 ml per injection site

- Dilution is 100 units per ml.

This dosage is recommended by the FDA and is within the accepted range recommended by Shriners Botox Task Force as well as the American Academy of Cerebral Palsy and Developmental Medicine. Although some authors have used higher doses of BTX-A, we believe that higher doses may weaken the muscles such as rehabilitation and early weight bearing might become a problem, thus defeating the purpose of the injections.

#### iii Dosage Calculation

Calculate maximum dose allowed according to child's weight. For example, a 40 kg patient could receive the maximum dose of 400 units.

#### iv: Number and location of Injection Sites

Calculate the number of injection sites according to the maximum dose distributed. Agent will be injected into the bellies of the muscle groups adjacent to the bone lengthened according to the following guidelines. Diagrams for anatomical location are included in study manual.

Femoral Lengthening:

Quadriceps muscle group: (max 200 Units)/50 Uper site = 4 sites

1. Vastus Medialis (1)

2. Vastus Lateralis (1)

3. Vastus Intermedius/Rectus Femoris (2)

Hamstrings muscle group: (max 200 Units)/50 Uper site = 4 sites

1. Medial Hamstrings (4)

Tibial Lengthening/Correction:

Gastrocsoleus Muscle group: (max 200 Units)/50 Uper site = 4 sites

1. Gastrocnemius (2)

2. Soleus (2)

Clubfoot correction:

Gastrocsoleus Muscle group (max 200 Units)/50 Uper site = 4 sites

1. Gastrocnemius (2)

2. Soleus (2)

### 7. Adverse Events

All serious adverse experiences will be reported. Minor adverse events and unusual complications encountered during the lengthening and or correction process will be carefully recorded. Side effects include a flu-like syndrome that is generally short-lived, dysphagia, and dry mouth. Unintended weakness of the muscle injected or of nearby muscles could also be a negative effect. Other side effects include muscle soreness, rash, headaches, light-headedness, fever, chills, hypertension, diarrhea and abdominal pain.

In the event of a serious adverse reaction to the treatment the patient will be treated accordingly depending on the reaction. The following events will cause immediate cessation of the study.

1. Death

2. Upper limb paralysis or weakness

3. Bulbar weakness

a. Blurred vision

b. Facial paralysis

c. Dysarthria

4. Anaphylactic reaction

5. Any severe ADR

Unblinding of the patient will be carried out immediately. The event will be analyzed in the same manner as any severe ADR. This will be reported to the appropriate agencies with the analysis, conclusions and recommendations. The study will then be re-instituted if given approval to continue. This patient will be categorized as a treatment failure and assigned (imputed) the most negative score on all outcomes.

### 8. Pain Management Protocol

Pain management (assessment and treatment) guidelines are described in the study manual. Post operative pain medication is delivered via either PCA or epidural up to 72 h post-op. Pain assessment will be done using a 10 point scale (either Faces Pain Scale revised or the Numerical Rating Scale) according to post-operative PCA protocols; hourly for first 4 hours, every 2 hours for next 8 hours, and every 4 hours once stable. After the PCA/epidural is discontinued, PRN medications will be ordered for pain control, nausea/vomiting, itching, and fever during hospitalization. Pain assessments will also be done whenever a patient receives a PRN medication. All of this data will be recorded in the patient's chart along with the medication dosages administered.

### 9. Post operative Activity and Rehabilitation Regimes

Patients will stay in hospital for 4–7 days following application of the external fixator and administration of the botox/placebo and until they are independent with the turnings. During this period patients will follow the pain management guidelines suggested for this group. All patients will also be seen by a physical therapist for a standardized exercise regime. During the distraction phase patients will be followed in clinic on a biweekly basis and during the consolidation phase on a monthly basis. The children will continue to be followed for three months after the external fixator is removed. Pain management, physical therapy, radiographs and adverse event assessment will continue during the entire lengthening process. Physical therapy and pain protocols are outlined in a study manual.

### 10. Data Safety and Monitoring Committee (DSMC)

A Data and Safety Monitoring Committee (DSMC), consisting of two experts in orthopedics and one biostatistician, will be constituted of experts with no vested interest in the trial. The DSMC will be responsible for supervision of the interim analysis, monitoring of the trial, and review of all adverse events. The study will be stopped if the interim analysis shows statistically significant results.

The study is blinded to the physician and assessors of outcomes. The pharmacist at each center is responsible to track each patient in the study, record which medication was injected, and the clinical staff will record any adverse effects noted. A standard data collection sheet will list each patient, the dosage given and any adverse outcomes. The information will be collected concurrently during the immediate post-injection period with each local co-investigator informed of any adverse effects. **For safety, the investigators at each participating center will have access to which medication is injected if treatment of an acute adverse event is necessary**. They will update the information to the PI on a weekly basis as needed. The PI and clinical staff are responsible for monitoring patient safety by tracking and collating the data from all participating centers. All adverse effects will be reported to the appropriate oversight committees (local IRB and FDA). The PI is responsible to communicate these findings and any changes in protocol as needed to the co-investigators.

### 11. Outcome Assessment

The outcome measures selected for the study relate directly to the specific aims of the project. The data collected will provide information on the pain scores, medication dosages, range of motion, flexibility, strength, mobility function, and quality of life of the patient population under study. Additional data will be collected on the patient demographics, musculoskeletal information, impact on the family during the distraction process, and motivation of the child. During the pilot study the participating hospitals finalized the selection of the outcome tools, the data collection forms of physiotherapy and nursing, as well as the timing of administration. The research coordinator at each site will ensure that the timeline is respected.

The measures will be administered pre-lengthening, pre-distraction, mid-distraction, end distraction, mid-consolidation, pre-frame removal and 3 months post-frame removal. Pain scores, medication dosages and compliance to physical therapy will be recorded daily during admission and throughout the distraction/consolidation process. A timeline as shown in Table 1 will indicate when the different variables will be measured and which tool is given at which phase of the process. The time required to complete the assessments is 20–30 minutes.

Standardized assessments chosen have been validated and are reliable. The outcome measures are described below.

The **Numerical Rating Scale (NRS**) is a reliable and valid tool that is widely used in pediatric centers as a measure of acute pain. The **Faces Pain Scale-Revised **is a reliable and valid pain scale for younger children. These tools will be used by nursing to assess postoperative pain during the first 5–7 days following surgery. The same scale will be used by physiotherapists to assess pain pre and post-physiotherapy treatment. In addition patients will be familiar with these scales and will use them to rate their pain in a Pain/Medication/PT compliance journal throughout the study. The NRS has been translated into several languages. Guidelines accompany both the NRS and Faces Pain Scale-Revised ensuring that they are administered in a uniform fashion [75].

**Medication dosages **are extracted from the inpatient medical record and recorded on custom data collection forms. The pain scores from the inpatient and outpatient period are also transcribed to this form.

The **Adolescent Pediatric Pain Tool (APPT) **is a three part tool which includes a visual analogue scale, a listing of 42 words that describe pain qualitatively and body diagrams to localize the pain. Its validity and reliability have previously been established. Furthermore, this tool was previously used in the only other study on pain during treatment of limb length discrepancy [15,76,77] The APPT will be administered at the key pre-determined intervals to measure the current, pain underlying the lengthening procedure and in particular during the distraction phase.

The **active and passive range of motion (ROM) **of involved joints will be measured using a goniometer according to the Shriners Hospital for Children Motion Analysis Lab ROM protocols in order to evaluate the effect of BTX-A on muscle spasm and muscle contracture (Specific aim 2). Measurement will be recorded by the treating physical therapist as of post-op day 2, continuing daily until discharge, weekly during distraction and monthly during consolidation. Standard flexibility tests using measurements of angles will also be recorded. **Strength **will be measured by active movement and by the extent of the quads lag at baseline and at 3 months post frame removal.

The **Gillette Functional Assessment Questionnaire (FAQ) **was developed by the Gillette Children's Specialty Healthcare to measure ambulation status. The pre-op version includes a 10-level parent report walking scale, need for assistive devices, and lists to describe limits to walking and additional activities commonly performed in the standing position. The walking scale only will be administered according to the timeline. The inter and intra rater reliability of the walking scale has previously been demonstrated as well it is sensitive to changes in orthopedic conditions.

The **Pediatric Quality of Life Inventory (PedsQL) **is a 23-item questionnaire developed by Dr. J. Varni at the Center for Child Health Outcomes at the Children's Hospital and Health Care Center, San Diego. According to the authors, the PedsQL measures health related quality of life in healthy children and children with health conditions. The generic module which consists of four domains: physical health, emotional, social, and school, as well the pain section (4 items) of the rheumatology module, will be administered to assess quality of life during the lengthening or correction process. The PedsQL has a parent and child version for five different age groups and so measures both perspectives of the impact of the procedure. It is self-administered and has been translated into French and Spanish. Completion requires 10 minutes. The PedsQL has proven reliability and validity and can distinguish between healthy children and those with health conditions. [78].

The **Impact on Family Scale (IOF) **is a 27-item inventory translated into French and Spanish that takes approximately 10 minutes to complete and measures a parent's perception of the effects of the child's ongoing health condition on family life. The IOF will measure the amount of stress and disequilibrium experienced by the family of the child undergoing the painful and long lengthening or correction. It will be administered at the end of the process. It is not disease specific and can be used with minimal training. The measure has been widely used since 1980 and has good face validity. It has recently undergone a revised scoring procedure and additional psychometric tests showing that it is a reliable and valid measure [79].

The **Dimensions of Motivation Questionnaire (DMQ-17) **was developed by Morgan, Maslin-Cole, Biringen and Harmon to measure motivation, a multi-faceted, intrinsic psychological force that stimulates an individual to attempt to master a skill or task that is at least moderately challenging for him. The DMQ was recently revised to include all ages of children. It consists of a 45-item questionnaire of 5 scales: object oriented persistence, social/symbolic persistence, gross motor persistence, mastery pleasure, and general competence. The DMQ will measure a child's underlying motivation to determine what role it may play in the outcome of lengthenings. The recent re-scoring of the DMQ has produced a conceptually stronger and psychometrically stronger questionnaire [80,81].

A **Pain**/**Medication/PT Compliance Journal **will record the amount of analgesic medication required daily (dosage, frequency, time), pain experienced and compliance to Physical Therapy home exercise program during the outpatient period. The family will be provided with a customized booklet for this purpose.

**Radiographs **will be obtained during the process to assess the amount of lengthening, state of new bone formation, quality of the regenerate, and development of osteopenia.

### 12. Standardization

A study manual was prepared based on the experience gained from the pilot work, which outlines the protocols for assessments; injection, therapy, and pain management. The manual was discussed at the face-to-face meetings in 2005. Consensus was reached on key points and it was revised in December 2005

Regular conference calls and an annual meeting are planned to further ensure consistency across sites.

Individual patient tracking sheets ensure that data collection is complete at each time point.

### 13. Data Management

All data will be kept confidential using a coding system unique to each hospital. With the input of a medical information specialist from headquarters, a collaborative research site will be established on the Shriners intranet system to facilitate an electronic interface for data collection and data transfer.

Pain scores and medication dosages will be extracted from the medical record and transferred to a customized data collection form available on the intranet site. Hospitals will send the data collection forms by courier to the project manager at the Montreal Hospital where trained personnel will enter all the data into the Access database. Paper copies of the questionnaires will record the pertinent scores and the completed questionnaires (identified by study number only) will be sent by courier to the lead hospital and treated similarly.

This database will include some validation processes to ensure the quality of the data. Any doubtful data will be checked with the person responsible at the site where it occurred, and changes to the database will be documented. A single person at the Lead hospital will do the data entry to ensure reliability of the process. The data analysis will be performed by bio-statistician consultants to the project.

### 14. Statistical Analysis

Descriptive statistics will be calculated for all outcome variables and all relevant baseline variables. The data will be presented in summary tables, graphs, and listings to give an overview of the outcome and efficacy findings. The mean, median, standard deviation, and range of each variable and its change from baseline, measured on a continuous scale, will be presented by treatment and visit. Frequency tables will be provided for categorical variables by treatment and visit.

There are three primary outcomes: pain measured by the FPS-r or NRS, requirements of pain medication, and the quality of life using the PedsQL. The primary analysis for the pain will be performed during the post-operative (5–10 days) period as well as the mid-distraction phase. The primary analysis for the pediatric quality of life will be performed at mid-distraction and the mid-consolidation phase before the removal of the fixator. The primary analysis will be conducted using T-tests for continuous variables and chi-square and Fisher exact tests for categorical variables.

An interim analysis will be performed when 75 patients (1/2 the estimated sample size) have been enrolled, using the Obrien-Fleming group-sequential stopping rule. [82,83]

#### Primary statistical hypotheses

• Pain scores will be less, in those who received BTX-A, during the 5–10 days post-operative period compared to those who received saline injections (Specific aim 1).

• Pain scores will be less, in those who received BTX-A, at mid-distraction compared to those who received saline injections (Specific aim 1).

• The total amount of narcotic pain medication during the 10 days post-operative will be lower in those patients who received BTX-A than in those who received saline injections.

• Quality of life scores during mid-distraction will be higher in those who received BTX-A compared to those who received saline injections (Specific aim 2).

• Range of motion measurements and mobility scores will be improved in those who received BTX-A compared to those who received saline injections (Specific aim 3).

Primary Outcome Measures: FPS-r/NRS, PedsQL, ROM

#### Secondary Analysis

Secondary longitudinal analyses will be carried out and include several time periods and compare additional variables as shown in the following table. Stratification by age or site of fixator or type of surgery (lengthening versus correction) will allow further analyses. T-tests and analyses by repeated measures will be used for the secondary analyses.

#### Sample size

The proposed sample size of 75 per group provides 80% power to detect an effect size of 0.45, with alpha = 0.05. The effect size of 0.45 corresponds to a difference of approximately 7.5 in the PedsQL (Ref Varni et al 2002, Varni et al 2003). This difference is smaller than that found in studies comparing children with chronic diseases to healthy children. The following table provides a range of options for sample size, using the PedsQL as an example. For a standard deviation (SD) of 15 and alpha (type I error) of 0.05, the power is as given in the first column for different values of delta (the detectable difference between the groups). The differences detected in the pilot work support the planned sample size.

## Discussion

The global aim of this proposal is to determine if BTX-A can alleviate the post-operative pain and improve the functional outcome of children undergoing limb lengthening or deformity correction.

We have decided to measure in the post op period both the total amount of pain as well as the total amount of narcotics since both parameters are interconnected. Some patients may have very little pain but on the other hand may have received large doses of narcotics, therefore it is important to measure and record both parameters.

The mobility variables, weight bearing, range of motion and level of ambulation are indicative of a higher level of participation. In addition, an increased level of mobility is associated with lengthenings that have fewer complications such as delayed healing.

Reduced pain and increased mobility are felt to contribute to an improved quality of life for children undergoing lengthening. Clearly, these key variables are linked during the long and painful process of distraction osteogenesis and botox may assist in initiating this process.

## Abbreviations

APPT Adolescent and Pediatric Pain Tool

BTX-A Botulinum Toxin A

CNS Central Nervous System

CRGP Calcitonin Gene Related Peptide

DMQ Dimensions of Motivation Questionnaire

DO Distraction Osteogenesis

DSMC Data Safety and Monitoring Committee

EMG Electromyography

FAQ Gillette Functional Assessment Questionnaire

FDA Food and Drug Administration

FPS-R Faces Pain Scale revised

HIPAA Health Information Privacy, Portability and Accountability Act

ICF International Classification of Functioning, Health and Disability

IOF Impact on Family Questionnaire

IRB Institutional Review Board

NRS Numerical Rating Scale

OR Operating Room

PedsQL Pediatric Quality of Life Inventory

PCA Patient controlled analgesic

PI Principal Investigator

PT Physical Therapy

ROM Range of Motion

QOL Quality of Life

## Compteting interests

The author(s) declare that they have no competing interests.

## Authors' contributions

RH: orthopaedic surgeon. Performed the limb lengthening surgery, participated in the design of the study as well as preparing the manuscript.

KM: Chief of OT. Coordinated the project, participated in the design of the study as well as preparing the manuscript.

JRG: Chief of PT: Responsible for all clinical measurements.

KT: Research Nurse Assistant. Participated in the design of the study.

ER: Orthopaedic surgeon. Performed the limb lengthening surgery, participated in the design and data collection of the study

MA: Orthopaedic Surgeon. Performed the limb lengthening surgery. Participated in attaining FDA approval for study.

RP: Performed sample size calculations and statistical plan.

AF: Anesthesiologist, consultant on pain management and measurement

WM: Orthopaedic surgeon. Performed the limb lengthening surgery, participated in the design and data collection of the study

JM: Orthopaedic surgeon. Performed the limb lengthening surgery, participated in the design and data collection of the study

UN: Orthopaedic surgeon. Performed the limb lengthening surgery, participated in the design and data collection of the study

All have authors read and approved the final manuscript
